# Vertical transmission of SARS-CoV-2: A systematic review

**DOI:** 10.1177/1753495X211038157

**Published:** 2022-06

**Authors:** Kristine Jeganathan, Anthea BM Paul

**Affiliations:** 1 12365 Faculty of Medicine, University of Ottawa, Canada; 2 Department of Family and Community Medicine, University of Toronto, Canada

**Keywords:** Neonate, pregnancy, newborn, COVID-19, vertical transmission, SARS-CoV-2

## Abstract

In this study, we discuss vertical transmission of SARS-CoV-2, and assess various maternal and neonatal outcomes based on the current evidence available. This systematic review using PRISMA guidelines revealed a total of 47 eligible studies describing 1188 SARS-CoV-2 positive pregnant women and 985 neonates for review. Utilizing the ‘Shah’s Classification System for Maternal-Fetal-Neonatal SARS-CoV-2 Intrauterine Infections’ by Shah et al., we found vertical transmission confirmed in 0.3% (*n* = 3), probable in 0.5% (*n* = 5), possible in 1.8% (*n* = 17), unlikely in 80.3% (724) and not infected in 17% (*n* = 153).

## Introduction

The COVID-19 outbreak was declared a global emergency on 30 January 2020 with an estimated 185 million infected individuals with 4 million attributed global deaths at the time of writing.^1,2^ Research is ongoing on the effects of COVID-19 on vulnerable populations, and there remains uncertainty regarding the clinical characteristics and vertical transmission potential of the SARS-CoV-2 infection in pregnancy. Although less common with viral than with bacterial infections, perinatal transmission of infections from mother to neonate is a significant cause of neonatal morbidity.^3^ For example, viral pneumonia is an important cause of neonatal morbidity and mortality in pregnancy, associated with premature rupture of membranes, preterm labour, intrauterine fetal death, intrauterine growth restriction and neonatal death.^4,5^ Perinatal neonatal infections can occur through three different routes: intrapartum transmission from vaginal secretions or tears, respiratory or droplet transmission from contact between the neonate and others or transmission across the placenta.^6^ There is minimal evidence discussing the likelihood of vertical transmission of SARS-CoV-2 with over-representation of case reports and case studies having suggested the possibility of intrauterine infection.^7,8^ This systematic review examines the literature on pregnant women who tested positive for SARS-CoV-2, and the outcomes of their neonates including whether vertical transmission occurred. Furthermore, we will discuss common maternal and neonatal symptoms and outcomes, as well as discuss the hypothesized mechanisms for intrauterine transmission.

## Methods

The protocol was designed and conducted in accordance with the 2009 PRISMA (Preferred Reporting Items for Systematic Reviews and Meta-analyses) guidelines.^9^ This systematic review includes case reports, case series, retrospective and prospective cohort studies of pregnant women diagnosed with COVID-19. Search strategy: the search strategy aimed to identify studies relevant to the likelihood of vertical transmission of COVID-19 in pregnancy. The electronic databases, Medline (1946–2020, OVID), Embase (1974–2020) and Scholar were searched on 9 October 2020. These databases were comprehensively searched to identify articles published in December 2019 up to September 2020, using Medical Subject Headings (MeSH) and keywords to pinpoint relevant articles. The following string of keywords and MeSH terms were used to identify relevant articles: (‘fetus*’ OR ‘neonate*’ OR ‘neonatal outcome*’ OR ‘pregnancy’ OR ‘newborn*’ OR ‘Gestation’ OR ‘maternal’ OR ‘mother’ OR ‘delivery’ OR ‘infant*’) AND (‘intrauterine transmission’ OR ‘vertical transmission’ OR ‘maternal-infant infection’ OR ‘Transplacental transmission’) AND (‘COVID-19’ OR ‘SARS-CoV-2’ OR ‘severe acute respiratory syndrome coronavirus 2’ OR ‘Coronavirus Disease 2019’ OR ‘Corona Virus’). The two independent reviewers (K.J. and A.P.), analyzed the articles individually, beginning with the title and abstract screening. Irrelevant articles that did not fit the inclusion criteria were removed and missed relevant articles were added from study references. After the full text review was complete, both authors extracted data from the selected articles and sorted them into tables.

## Inclusion criteria

All selected studies that were analyzed in this systematic review were retrospective or prospective cohort studies, case reports or case series. Two independent reviewers also evaluated the quality score of each article using the Mayo Evidence-Based Practice Center tool, for evaluating the quality of case reports and case series.^10^ In order to be included in this study, the following inclusion criteria was applied: (1) Pregnant woman who tested positive for SARS-CoV-2 using blood or nasopharyngeal swabs and severe acute respiratory syndrome coronavirus 2 viral RNA test. (2) The neonate of the reporting mother must have been tested for SARS-CoV-2. The method of testing for neonates was reverse transcriptase polymerase chain reaction (RT-PCR) analysis of naso-pharyngeal swab. (3) Must be an original article, written in, or translated to English. (4) The outcome of the study was clearly outlined, either demonstrating evidence for or against vertical transmission of SARS-CoV-2. (5) The methodological quality of each article must be higher than or equal to 3 (using the Mayo Evidence-Based Practice Center tool, for evaluating the quality of case reports and case series).^10^ If a study was a non-original article, non-peer reviewed, written in any language other than English (that could not be translated) and/or used animal models it was excluded from this review. Furthermore, if an article received a quality score lower than 3, it was excluded from this review. We used the Shah classification system to help distinguish between an intrauterine and nonintrauterine (intrapartum or environmental) mode of transmission for an infected neonate.^11^ Each study was analyzed using the Shah et al. classification system consisting of five categories for the likelihood of intrauterine infections. In order to be classified as a ‘confirmed’ intrauterine infection, there must be detection of SARS-CoV-2 in the umbilical cord blood, in neonatal blood within 12 h of birth, or in the amniotic fluid. A ‘probable’ infection involved detection of SARS-CoV-2 in the neonate’s NP swab immediately after birth and detection of the virus on the fetal side of the placenta. A ‘possible’ infection involved no detection of SARS-CoV-2 in the neonate’s NP swab, and presence of IgM antibodies in neonate’s blood or placental tissue immediately after birth. An ‘unlikely’ infection involved no detection of SARS-CoV-2 in the neonate’s NP swab at birth or in the neonate's blood, and IgM was not measured. Finally, the ‘not infected’ category involves no detection of SARS-CoV-2 in the neonate’s NP swab, cord blood, amniotic fluid, placenta and no IgM antibodies present in neonate. If a case did not meet the criteria in this classification system, it was not included in the totals and was indicated in the table with an asterisk. In our study, if a neonate was not tested immediately after birth, but tested positive within 24 h, and proper isolation protocols were in place, we placed these cases in the ‘possible’ intrauterine infection category. These studies are included in Table 1 and marked with an asterisk (*). These cases were not included when calculating totals and the percentages of intrauterine infection risk.

**Table 1.** Characteristics of articles included in the systematic review.

Author	Type of study	Country	Number of women testing positive for SARS-CoV-2	Number of neonates tested for SARS-CoV-2	Evidence to support vertical transmission?	Shah’s Classification System for Maternal-Fetal-Neonatal SARS-CoV-2 Intrauterine Infections^11^
Abasse et al.^12^	Case report	France	1	1	(+)	Unclassified – Possible (1)*
Algarroba et al.^13^	Case report	United States	1	1	(+)	Possible (1)
Alzamora et al.^14^	Case report	Peru	1	1	(+)	Unclassified – Possible(1)*
Blauvelt et al.^15^	Case report	United States	1	1	(–)	Unlikely (1)
Cao et al.^16^	Retrospective review	China	10	11	(–)	Unlikely (11)
Chen et al.^17^	Retrospective review	China	9	9	(–)	Not infected (9)
Demirjian et al.^18^	Case report	United Kingdom	1	1	(+)	Unlikely (1)
Dong et al.^19^	Case report	China	1	1	(+)	Possible (1)
Fan et al.^20^	Case series	China	2	2	(–)	Not infected (2)
Fenizia et al.^21^	Prospective cohort study	Italy	31	31	(+)	Confirmed (1)
						Probable (1)
						Not infected (29)
Gidlöf et al.^22^	Case report	Sweden	1	2	(–)	Unlikely (2)
Govind et al.^23^	Case series	United Kingdom	9	9	(+)	Possible (1)
						Unlikely (8)
Hu et al.^24^	Case report	China	7	7	(+)	Unclassified – Possible (1)*
						Unlikely (6)
Kalafat et al.^25^	Case report	Turkey	1	1	(–)	Not infected (1)
Khan et al.^26^	Case report	China	3	3	(–)	Not infected (3)
Khan et al.^27^	Case series	China	17	17	(–)	Unlikely (17)
Kirtsman et al.^28^	Case report	Canada	1	1	(+)	Probable (1)
Knight et al.^29^	Prospective cohort study	UK	427	265	(+)	Unclassified – Probable (6)*
						Possible (6)
						Unlikely (253)
Lang et al.^30^	Case report	China	1	1	(–)	Not infected (1)
Li et al.^31^	Case report	China	1	1	(–)	Not infected (1)
Liao et al.^32^	Retrospective cohort study	China	10	10	(–)	Unlikely (10)
Liu et al.^33^	Prospective study	China	19	19	(–)	Unlikely (19)
Lowe et al.^34^	Case report	Australia	1	1	(-)	Unlikely (1)
Lu et al.^35^	Case report	China	1	1	(-)	Unlikely (1)
Marzollo et al.^36^	Case report	Italy	1	1	(+)	Unclassified – Possible (1)*
Nie et al.^37^	Retrospective review	China	33	26	(+)	Unlikely (1)
						Not infected (25)
Oncel et al.^38^	Multicentre cohort study	Turkey	125	125	(+)	Unlikely (125)
Patanè et al.^39^	Retrospective review	Italy	22	22	(+)	Probable (2)
						Unlikely (20)
Patil et al.^40^	Retrospective cross-sectional study	United States	45	45	(+)	Possible (2)
						Unlikely (43)
Penfield et al.^41^	Retrospective Review	United States	11	11	(+)	Possible (3)
						Not infected (8)
Peng et al.^42^	Case report	China	1	1	(–)	Not infected (1)
Pereira et al.^43^	Retrospective review	Spain	60	23	(–)	Unlikely (17)
						Not infected (6)
Pierce-Williams et al.^44^	Retrospective cohort study	United States	64	33	(–)	Unlikely (33)
Sisman et al.^45^	Case report	United States	1	1	(+)	Confirmed (1)
Vivanti et al.^46^	Case report	France	1	1	(+)	Confirmed (1)
Wang et al.^47^	Case report	China	1	1	(+)	Unlikely (1)
Wu et al.^48^	Retrospective cohort Study	China	29	30	(+)	Unclassified – Possible (4)*
						Unlikely (1)
						Not infected (25)
Xiong et al.^49^	Case report	China	1	1	(–)	Not infected (1)
Yan et al.^50^	Retrospective Review	China	116	86	(–)	Unlikely (76)
						Not infected (10)
Yang et al.^51^	Retrospective review	China	7	7	(–)	Unlikely (7)
Yang et al.^52^	Prospective study	China	27	24	(+)	Unlikely (24)
Yin et al.^53^	Retrospective cohort Study	China	31	31	(–)	Not infected (31)
Yu et al.^54^	Retrospective study	China	7	3	(+)	Unlikely (3)
Zamaniyan et al.^55^	Case report	Iran	1	1	(+)	Probable (1)
Zeng et al.^56^	Cohort study	China	33	33	(+)	Unclassified – Possible (3)*
						Unlikely (30)
Zeng et al.^57^	Retrospective review	China	6	6	(+)	Possible (3)
						Unlikely (3)
Zhu et al.^58^	Case series	China	9	10	(–)	Unlikely (10)

*These cases of infection fulfil the inclusion criteria of the Shah classification system, and therefore were omitted from final calculations.

## Results

**Figure 1.**
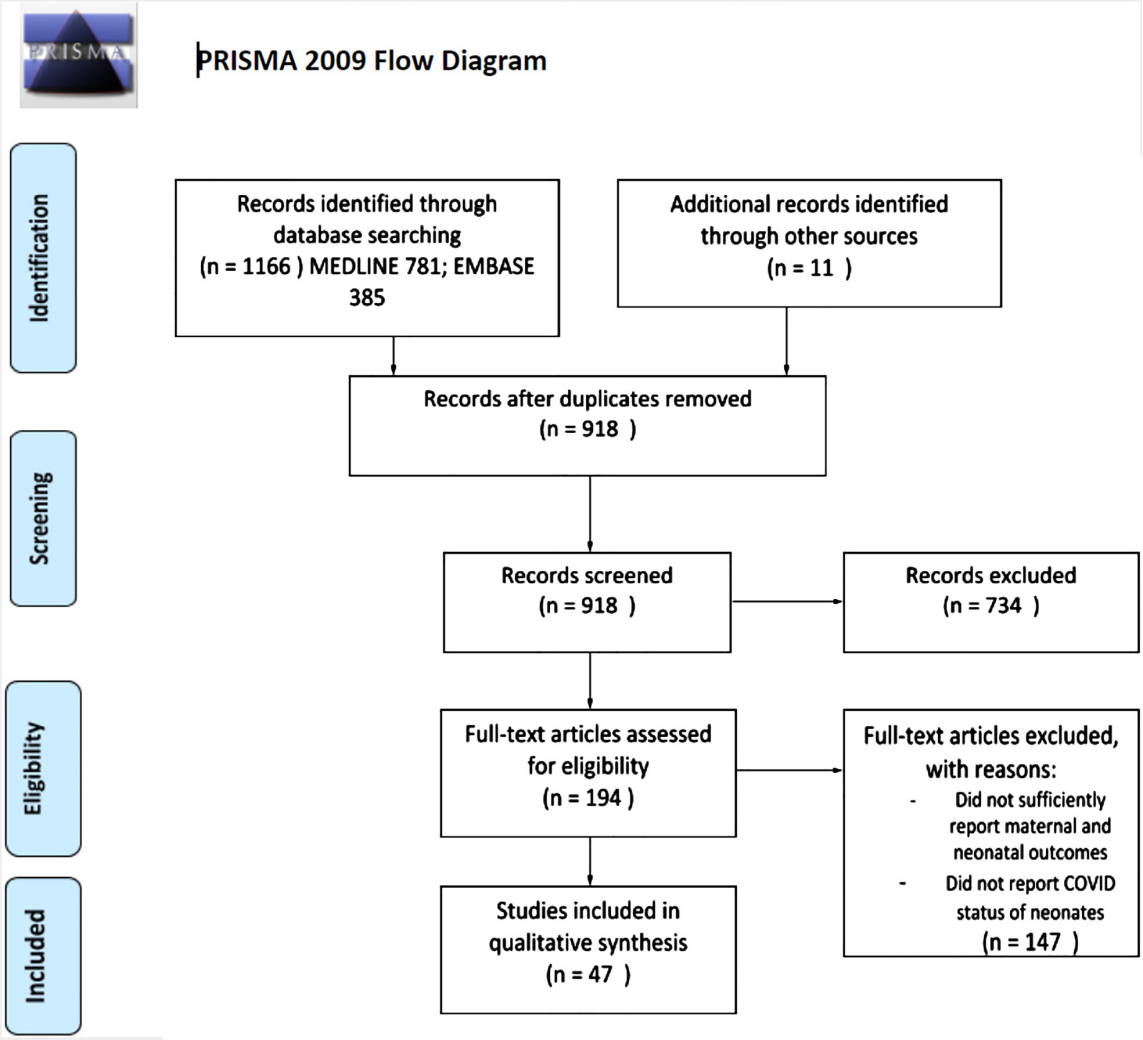
Study selection flow chart. The study selection process was done in accordance with the PRISMA (Preferred Reporting Items for Systematic Reviews and Meta-analyses) guidelines. The search strategy began with 1166 articles and was then narrowed down to 47 articles based on title/abstract scan and full-text screening. ‘The PRISMA Statement and the PRISMA Explanation and Elaboration document are distributed under the terms of the Creative Commons Attribution License, which permits unrestricted use, distribution, and reproduction in any medium, provided the original author and source are credited’.^9^

**Figure 2.**
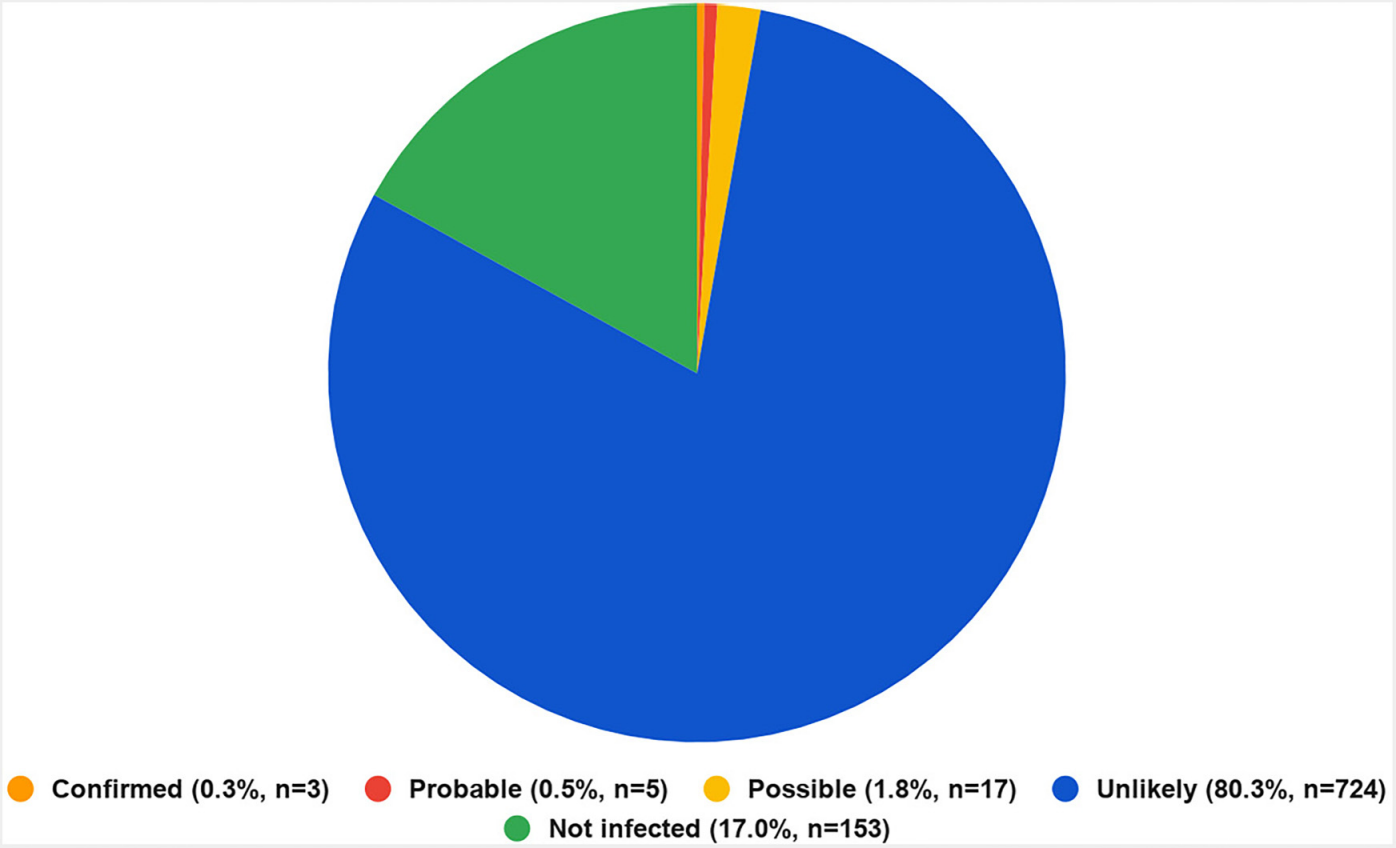
Distribution of neonatal infections using Shah's Classification System for Maternal-Fetal-Neonatal SARS-CoV-2 Intrauterine Infections.

A total of 47 eligible studies were selected, describing 1188 infected pregnant women and 985 tested neonates. In our review, we identified five studies that reported cases that did not meet the inclusion criteria for the Shah classification system. All five studies tested the neonate for SARS-CoV-2 12 h of birth, rather than immediately after. The project flow chart is illustrated in Figure 1, and study characteristics are described in more detail in Table 1. A large proportion of articles included in this review reported cases where intrauterine infection either did not occur (‘not infected’) (17.0%, *n* = 153), or was ‘unlikely’ (80.3%, *n* = 724) to have occurred (Figure 2). A considerable number of articles however, reported cases of confirmed (0.3%, *n* = 3), probable (0.5%, *n* = 5) or possible (1.8%, *n* = 17) intrauterine infection of SARS-CoV-2.

## Symptoms and outcomes of COVID-19 infected pregnant women

Our review identified a total of 1188 pregnant women that tested positive for SARS-CoV-2. The full description of maternal symptoms and outcomes are described in Supplementary Table 2. All of the women in our review were tested for SARS-CoV-2 during their third trimester of pregnancy. The most common presenting symptoms were fever (41.4%, *n* = 492), cough (31.9%, *n* = 379) and dyspnea/shortness of breath (18%, *n* = 215). Some common atypical SARS-CoV-2 symptoms in these women were diarrhoea (3.7%, *n* = 44), myalgia (5.3%, *n* = 64), abdominal pain (<1%, *n* = 7), headache (4.7%, *n* = 56), fatigue (6.8%, *n* = 81) and sore throat (4.8%, *n* = 57). A small proportion of women (11.7%, *n* = 139) were asymptomatic at the time of presentation but received a SARS-CoV-2 test due to recent exposure to a SARS-CoV-2-positive family member. The most common computed tomography scan findings were bilateral ground-glass opacities. There were unfortunately seven cases of maternal death, 22 ICU admissions requiring mechanical ventilation. For the majority of pregnant women, however, the outcomes were favourable, leading to discharge from hospital shortly after delivery. A small proportion of women studied (*n* = 29) were positive for the presence of SARS-CoV-2 in amniotic fluid, placental tissue or cord blood. Testing demonstrated placental positivity in 5 of 8 tested, cord blood was positive in 3 of the 22 tested and amniotic fluid was positive in 2 of the 20 tested.

## Infected neonatal symptoms and outcomes

Our review identified a total of 44 out of 919 (4.79%) neonates who tested positive for SARS-CoV-2 shortly after birth. The full description of infected neonatal symptoms and outcomes are described in Supplementary Table 3. The most common symptoms were respiratory distress (36.3%, *n* = 16), fever (1.8% *n* = 8), and feeding difficulties (20%, *n* = 9). A proportion of the neonates tested positive at birth 25% (*n* = 11), whereas a small proportion of neonates (6.8% *n* = 3) tested negative at birth and subsequently tested positive. The remainder of neonates (9/44) were tested at greater than 24 h after birth and tested positive. Of the total, in seven neonates the timing of the test was not reported. The 1 min Apgar scores of these neonates ranged from 2 to 10, with the median score being 8. The 5 min Apgar scores ranged from 4 to 10, with the median score being 9. The median 10 min Apgar score was 9. The majority of SARS-CoV-2 positive neonates (72.7%, 32/44) were delivered by caesarean section. Most neonates were immediately admitted to the NICU once they were symptomatic or had a positive RT-PCR test for SARS-CoV-2. Many neonates were treated with antibiotics such as ampicillin and gentamicin, and 15% (*n* = 7) required ICU admission with mechanical ventilation. Overall, however, all neonates had favourable outcomes and were discharged from hospital. The maximum time until discharge in our study was 50 days after birth.

## Discussion

To the best of our knowledge, this is the first systematic review on the likelihood of vertical transmission of SARS-CoV-2, which uses the ‘Shah's Classification System for Maternal-Fetal-Neonatal SARS-CoV-2 Infections’ classification system for each selected article.^11^ Notably other found systemic reviews on this subject were reviewed and included in this article.

We noted that among the neonates that tested positive for SARS-CoV-2, the majority of deliveries reported in these studies were through caesarean section, with rigorous infection control measures. Therefore, the mode of SARS-CoV-2 transmission would exclude through vaginal secretions but could still involve transmission through tears, respiratory or droplet transmission from contact between the neonate and others or alternatively through vertical transmission. This could provide evidence for transplacental transmission of the virus, although a transcervical route of infection cannot be completely ruled out.^24^ Studies also directly visualized the placenta of SARS-CoV-2-positive mothers shortly after delivery, with some showing evidence of vertical transmission. Govind et al.^23^ used transmission electron microscopy to identify virions invading the syncytiotrophoblasts in the placenta villi. Another study by Dong et al.^19^ found elevated IgM and IgG antibodies against SARS-CoV-2 in neonatal blood. IgM antibodies are not able to cross the placental barrier, therefore this study suggests that the neonates developed these antibodies after being exposed to SARS-CoV-2 in-utero.^57^

What is the mechanism for transplacental transmission of SARS-CoV-2?

The exact mechanism of SARS-CoV-2 intrauterine infection is still unknown. In order for transplacental transmission to occur, the SARS-CoV-2 virus must first be circulating in the bloodstream of the infected pregnant woman.^59^ It is proposed the virus will then invade uterine arterioles to cross into the fetal side of the placenta; the virus will then reach the chorionic villus and circulate in the fetus. Current research suggests that SARS-CoV-2 infection occurs through activation of the angiotensin-converting enzyme receptor on the surface of cells.

It is hypothesized that the likelihood of transplacental transmission of SARS-CoV-2 is higher, as gestational age increases. This is consequently thought to be contributed to the increasingly expressed angiotensin-converting enzyme 2 (ACE-2) receptors on the placenta closer to the end of gestation.^60^ Furthermore, animal models have demonstrated that ACE-2 receptor expression reaches its peak near the end of gestation.^31^ Furthermore, previous studies have reported that expression of ACE-2 receptors on human placenta is variable between women. This could support the discussion as to why vertical transmission is rare and variable between SARS CoV-2 positive individuals.^61^

Previous studies have stated that in order for transplacental transmission to occur, there must be a high viral load and viral replication level in the maternal blood.^49^ Viremia of SARS-CoV-2 is rare in infected adults. Some articles have hypothesized that a high viral load in combination with extensive inflammation can lead to viremia.^18^ It has been suggested that there may also be a correlation between the time/duration of viral exposure in-utero, and neonatal SARS-CoV-2 status. A longer duration of viral exposure may lead to an increased likelihood of neonatal infection.^38^ However, there is a paucity of robust evidence for this correlation, and future study is required.

Some previous studies have proposed that various comorbidities may affect the likelihood of intrauterine transmission.^22^ Furthermore, one study reports that there is no correlation between severity of disease progression and likelihood of vertical transmission. In this study, one mother presented with mild COVID symptoms, while the other had severe symptoms, and vertical transmission was seen in both cases.^16^ Our review suggests that severity of disease progression does not increase the risk of vertical transmission.

Is a SARS-CoV-2 diagnosis an indication for a caesarean section?

As previously discussed, the majority of infected neonates in this review were delivered through caesarean delivery. In many of these cases, the infected mothers had other indications such as preeclampsia, history of caesarean sections and fetal distress.^17^ Numerous studies have demonstrated that SARS-CoV-2 has not been found in vaginal secretions during pregnancy and delivery. Therefore, the current obstetric guidelines indicate vaginal delivery is safe.^43^ Some studies in our review demonstrated that with vaginal delivery, there were no cases of intrapartum SARS-CoV-2 transmission. One study showed that 78% of the infected mothers gave birth through vaginal delivery, and none of the neonates tested positive for SARS-CoV-2.^43^ A maternal SARS-CoV-2 diagnosis alone appears to not be an indication for Caesarean delivery to prevent SARS-CoV-2 in the neonate, however, isolation of the newborn and the postpartum mother is strongly recommended to prevent environmental transmission.

As discussed earlier, most outcomes of pregnant women that tested positive for SARS-CoV-2 were favourable. In some cases, however, some women progressed to critical disease states, sometimes leading to maternal death. For this reason, it is important to diagnose and treat SARS-CoV-2 in pregnant women as early as possible. Some studies emphasize the importance of chest computerised tomography (CT) as well, to detect a latent SARS-CoV-2 infection of a pregnant woman.^16^ The chest CT will often show bilateral ground-glass opacities. Regarding symptoms and outcomes of neonates infected with SARS-CoV-2, our review suggests that outcomes are favourable. We reported no fetal deaths or severe adverse outcomes. Although there was a considerable number of infected neonates that were asymptomatic shortly after birth, some of these neonates later developed severe pneumonia in hospital.^12^ Many studies emphasized the need for close follow-up of asymptomatic neonates and repeat SARS-CoV-2 testing, in order to ensure timely treatment of infected neonates. Specifically, it is suggested that neonates should be tested immediately at birth, and 72 h after. Furthermore, cord blood, placental specimens and amniotic fluid should also be tested. Timing of testing is very important to distinguish between intrauterine, intrapartum and environment mode of transmission.^28^

### Limitations of this systematic review

The first limitation for this systematic review is that the majority of selected studies were case reports and case series, due to the scarcity of data on this topic. These types of studies are considered the lowest quality evidence on the evidence pyramid.^11^ This methodology also limits the amount of data the reviewers were able to collect and analyze. Furthermore, for many of the studies included in this review, RT-PCR of NP swab specimens was solely used to diagnose SARS-CoV-2 in neonates. Although this is the gold stand for testing, it may not immediately capture positive tests in neonates, especially if the route of transmission is hematogenous.^41^ Therefore in some cases, some neonates that initially tested negative immediately after birth, then tested positive a few days later. Another limitation to these results is that many of the studies analyzed in this systematic review solely describe the clinical course of infection of these women infected in their third trimester. This is due to the fact that there is scarce information on the implication if infection occurs in the first or second trimester. Possible vertical transmission should be evaluated in all stages of pregnancy rather than solely reviewing third-trimester cases. Furthermore, many investigators did not test important specimens such as cord blood, amniotic fluid and placenta for the virus. Therefore, some articles that we classified as probable or possible cases of intrauterine transmission may actually be confirmed cases. In order to acquire more reliable results on the mode of transmission, hospitals should improve access to the molecular and antibody testing of cord blood, amniotic fluid and placental samples. This would allow researchers to truly distinguish the mode and mechanism of transmission of neonatal SARS-CoV-2 infection.^45^ There are also some limitations to the Shah criteria itself. The Shah criteria consider the presence of SARS-CoV-2 in umbilical cord blood, amniotic fluid and/or neonatal blood as a confirmative criterion of intrauterine infection. This, however, is risky to consider as a confirmative criterion because it does not take into account the possibility of contamination of these samples, especially when these samples are obtained at delivery. Most authors of the papers analyzed in this review did not specifically describe the mode in which the samples were collected, therefore this implies a risk of bias important to note with this classification system.

## Conclusion

In conclusion, safety measures should be enforced during delivery to prevent intrapartum transmission of SARS-CoV-2 from mother to neonate. Further high-quality studies are required to establish whether SARS-CoV-2 is vertically transmitted. This study reviewed pregnant women with SARS-CoV-2 in the last trimester of their pregnancy. Numerous studies describe the clinical course of infection of these women infected in their third trimester, however, there is scarce information on the implication if infection occurs in the first or second trimester. We suggest that an international multi-centre, prospective cohort study be performed to further understand the risk of vertical transmission of SARS-CoV-2 at all stages of pregnancy. Understanding the risk of vertical transmission of SARS-CoV-2 is crucial in order to establish standardized guidelines for obstetrical care for women infected with this virus around the world.

## Supplementary Material

Supplementary material
