# Supplementary material for: Vertical transmission of SARS-CoV-2: A systematic review
Source: Obstet Med. 2021 Aug 30;15(2):91–8. doi: 10.1177/1753495X211038157 (PMC9247633; doi:10.1177/1753495X211038157)
Supplement: sj-pdf-1-obm-10.1177_1753495X211038157 - Supplemental material for Vertical transmission of SARS-CoV-2: A systematic review [file sj-pdf-1-obm-10.1177_1753495X211038157.pdf]

Table 2. Maternal presenting symptoms and outcomes (of women that tested positive for).

Author	Gestational age at presentation	Gestational age at delivery	Presenting symptoms	Complications	Shah's Classification System for Maternal-Fetal-Neonatal SARS-CoV-2 Intrauterine Infections[11]
Abasse et al.[12]	33 weeks	33 weeks	• Cough<br>• Dyspnoea	• Preterm labour	Unclassified – Possible (1)*
Algarroba et al.[13]	28+4 weeks	28+4 weeks	• Cough<br>• Dyspnoea<br>• Hypoxia	• Sepsis pneumonia<br>• Metabolic acidosis<br>• Preterm labour	Possible (1)
Alzamora et al.[14]	33 weeks	33 weeks	• General malaise<br>• Fatigue<br>• Low-grade fever<br>• Dyspnoea	• Metabolic acidosis<br>• Preterm labour	Unclassified – Possible (1)*
Blauvelt et al.[15]	28 weeks	28 weeks	• Malaise<br>• Fever<br>• Myalgias<br>• Cough<br>• Dyspnoea	• ICU admission requiring mechanical ventilation<br>• Preterm labour	Unlikely (1)
Cao et al.[16]	33+6 to 40+5 weeks	3 late preterm	• Fever (2/10)<br>• Cough (1/10)<br>• Chest tightness (1/10)	• Preterm labour (3/10)	Unlikely (11)
Chen et al.[17]	36 weeks to 39+4 weeks	4 late preterm (all beyond 36 weeks)	• Fever (7/9)<br>• Cough (4/9)<br>• Myalgia (3/9)<br>• Sore throat (2/9)<br>• Malaise (2/9)<br>• Dyspnoea (1/9)	• Preterm labour (4/9)	Not infected (9)
Demirjian et al.[18]	38+5 weeks	39+5 weeks	• Fever<br>• Dyspnoea	• ICU admission requiring mechanical ventilation	Unlikely (1)
Dong et al.[19]	34+2 weeks	38 weeks	• Fever<br>• Nasal congestion<br>• Dyspnoea	• Preterm labour	Possible (1)
Fan et al.[20]	36 weeks and 37 weeks	37 weeks and 36 weeks	• Fever (1/2)<br>• Chills (1/2)<br>• Nasal congestion (2/2)<br>• Sore throat (1/2)	• Preterm labour (½)	Not infected (2)
Fenizia et al.[21]	33+6 weeks to 38 weeks	Not reported	• Not reported	• ICU admission required for mechanical ventilation (1/31)<br>• Interstitial pneumonia (14/31)<br>• Preterm delivery (1/31)<br>• C-section due to severe COVID-19 illness (3/31)<br>• Induction of delivery due to COVID-19 (6/31)	Confirmed (1)<br>Probable (1)<br>Not infected (29)
Gidlöf et al.[22]	36+2 weeks	36+2 weeks	• Headache<br>• Hoarseness<br>• Malaise	• Preeclampsia<br>• Preterm labour	Unlikely (2)
Govind et al.[23]	39 weeks+	39 weeks	• Anosmia (7/9)<br>• Cough (8/9)<br>• Myalgia (5/9)<br>• Fever (4/9)		Possible (1)<br>Unlikely (8)

(continued)

Table 2. Continued.

Author	Gestational age at presentation	Gestational age at delivery	Presenting symptoms	Complications	Shah's Classification System for Maternal-Fetal-Neonatal SARS-CoV-2 Intrauterine Infections[11]
			• Sore throat (4/9)<br>• Lethargy (6/9)<br>• Dyspnoea (4/9)		
Hu et al.[24]	Not reported	Not reported	• Low grade fever (4/7)<br>• Cough (2/7)<br>• Diarrhoea (1/7)		Unclassified – Possible (1)*<br>Unlikely (6)
Kalafat et al.[25]	35+3 weeks	36 weeks	• Cough<br>• Dyspnoea	• ICU admission required for mechanical ventilation<br>• Preterm labour	Not infected (1)
Khan et al.[26]	34+6 to 38+2 weeks	34+6 weeks to 39 +1 weeks	• Fever (2/3)<br>• Cough (3/3)<br>• Chest tightness (1/3)	• Preterm labour	Not infected (3)
Khan et al.[27]	35 weeks to 41 weeks	35+5 to 41 (average=38.1 weeks)	• Fever (3/17)<br>• Cough (6/17)<br>• Diarrhoea (3/17)<br>• Dyspnoea (2/17)<br>• Nasal congestion (2/17)<br>• Sputum (1/17)	• Preterm labour (5/17)	Unlikely (17)
Kirtsman et al.[28]	35+5 weeks	35+5 weeks	• Myalgia<br>• Decreased appetite<br>• Cough<br>• Fever	• Preterm labour	Probable (1)
Knight et al.[29]	Average=34 weeks	Median=38 weeks	• Fever (275/427)<br>• Cough (230/427)<br>• Dyspnoea (155/427)<br>• Lethargy (60/427)<br>• Headache (55/427)<br>• joint pain (50/427)<br>• sore throat (45/427)<br>• Diarrhoea (20/427)	• ICU admission required for mechanical ventilation (10/427)<br>• Interstitial pneumonia (24/427)<br>• Maternal death (1)	Unclassified – Probable (6)*<br>Possible (6)<br>Unlikely (253)
Lang et al.[30]	35+2 weeks	35+2 weeks	• Cough	• Preterm labour	Not infected (1)
Li et al.[31]	35 weeks	35 + 4 weeks	• Cough	• Preterm labour	Not infected (1)
Liao et al.[32]	31+1 weeks to 42 weeks	31+1 weeks to 42 weeks	• Fever (5/10)<br>• Cough (3/10)<br>• Sore throat (1/17)<br>• Chest pain (1/10)	• Preterm labour	Unlikely (10)
Liu et al.[33]	35+2 weeks to 41+2 weeks	38.6±1.5 weeks	• Fever (11/19)<br>• Cough (5/19)<br>• Dyspnoea (5/19)<br>• Diarrhoea (2/19)	• Preterm labour (2/19)	Unlikely (19)
Lowe et al.[34]	40 weeks	40+2 weeks	• Asymptomatic		Unlikely (1)
Lu et al.[35]	38 weeks	38 +2 weeks	• Asymptomatic		Unlikely (1)
Marzollo et al.[36]	38 weeks	38 weeks	• Fever	• Severe thrombocytopenia	Unclassified – Possible (1)*
Nie et al.[37]	28+weeks to 40 weeks	Not reported	• Fever (21/33)<br>• Cough (13/33)<br>• Fatigue (7/33)<br>• Dyspnoea (7/33)	• Preterm labour (10/33)	Unlikely (1)<br>Not infected (25)
Oncel et al.[38]	Not reported	26–38 weeks	• Not reported	• ICU admission requiring mechanical ventilation (8/125)	Unlikely (125)

(continued)

Table 2. Continued.

Author	Gestational age at presentation	Gestational age at delivery	Presenting symptoms	Complications	Shah's Classification System for Maternal-Fetal-Neonatal SARS-CoV-2 Intrauterine Infections[11]
				• Preterm labour (33/125)<br>• Maternal death (6/125)	
Patanè et al.[39]	35+1 to 37+6 weeks	35+1 to 37+6 weeks	• Fever (2)<br>• Cough (2)	• Preterm labour (½)	Probable (2)<br>Unlikely (20)
Patil et al.[40]	30+5 weeks to 41 weeks	30+5 weeks to 41 weeks	• Fever (15/45)<br>• Cough (16/45)<br>• Sore throat (4/45)<br>• Shortness of breath (6/45)	• ICU admission requiring mechanical ventilation (2/45)<br>• Preterm labour (4/45)	Possible (2)<br>Unlikely (43)
Penfield et al.[41]	26+5 weeks to 41+3 weeks	26+5 weeks to 41+3 weeks	• Not reported	• Preterm labour (3/9)	Possible (3)<br>Not infected (8)
Peng et al.[42]	35+3 weeks	36 weeks	• Fever<br>• Fatigue<br>• Dyspnoea	Preterm labour	Not infected (1)
Pereira et al.[43]	Median=32 weeks	Median=32 weeks	• Asymptomatic (15/60)<br>• Dyspnoea (17/60)<br>• Fever (34/60)<br>• Cough (34/60)	• ICU admission required for mechanical ventilation (1/60)<br>• Preterm labour (2/60)	Unlikely (17)<br>Not infected (6)
Pierce-Williams et al.[44]	Mean=30 + 6 weeks	Mean=30+6 weeks	• Not reported	• Maternal cardiac arrest (1/17)<br>• Preterm labour (15/17)	Unlikely (33)
Sisman et al.[45]	34 weeks	34 weeks	• Fever<br>• Diarrhea<br>• Back pain	• Preterm labour	Confirmed (1)
Vivanti et al.[46]	35+2 weeks	35+5 weeks	• Fever<br>• Cough	• Preterm labour	Confirmed (1)
Wang et al.[47]	40 weeks	40 weeks	• Fever<br>• Abdominal pain<br>• Vaginal bleeding		Unlikely (1)
Wu et al.[48]	35 weeks to 40 weeks	35 weeks to 40 weeks	• Fever (8/29)<br>• Cough (9/29)<br>• Diarrhoea (2/29)<br>• Dyspnoea (3/29)<br>• Asymptomatic (15/29)	• Preterm labour (3/29)	Unclassified – Possible (4)*<br>Unlikely (1)<br>Not infected (25)
Xiong et al.[49]	33+1 weeks	38+4 weeks	• Fever<br>• Cough<br>• Chills		Not infected (1)
Yan et al.[50]	Median = 38 weeks	Median = 38 weeks	• Fever (59/116)<br>• Cough (33/116)<br>• Asymptomatic (27/116)	• ICU admission requiring mechanical ventilation (2/116)<br>• Preterm labour (6/116)	Unlikely (76)<br>Not infected (10)
Yang et al.[51]	36 weeks to 37 weeks	36 weeks to 37 weeks	• Fever (6/7)<br>• Cough (6/7)<br>• Abdominal pain (6/7)<br>• Diarrhoea (6/7)	• Preeclampsia (2/7)<br>• Preterm labour (4/7)	Unlikely (7)
Yang et al.[52]	30 weeks to 40 weeks	30 weeks to 40 weeks	• Fever (13/27)<br>• Cough (9/27)<br>• Vomiting (1/27)	• Preterm labour (1/27)	Unlikely (24)

(continued)

Table 2. Continued.

Author	Gestational age at presentation	Gestational age at delivery	Presenting symptoms	Complications	Shah's Classification System for Maternal-Fetal-Neonatal SARS-CoV-2 Intrauterine Infections[11]
Yin et al.[53]	Mean=35 weeks	Mean=35 weeks	• Fever (17/31)<br>• Cough (15/31)<br>• Fatigue (6/31)<br>• Dyspnoea (8/31)<br>• Diarrhoea (2/31)<br>• Myalgia (3/ 31)	• Preterm labour (5/31)	Not infected (31)
Yu et al.[54]	37 weeks to 41+2 weeks	Mean=39+2 days	• Fever (6/7)<br>• Cough (1/7)<br>• Dyspnoea (1/7)<br>• Diarrhoea (6/7)		Unlikely (3)
Zamaniyan et al.[55]	32 weeks	33 weeks	• Dyspnoea<br>• Myalgia<br>• Anorexia<br>• Nausea<br>• Cough<br>• Fever	• ICU admission requiring mechanical ventilation<br>• Premature labour<br>• Maternal death	Probable (1)
Zeng et al.[56]	31 weeks to 40 weeks	31 weeks to 40 weeks	• Fever (8/33)<br>• Dyspnoea (4/33)<br>• Cough (10/33)	• Preterm labour (3/33)	Unclassified – Possible (3)*<br>Unlikely (30)
Zeng et al.[57]	>30 weeks	Not reported	Not reported		Possible (3)<br>Unlikely (3)
Zhu et al.[58]	>34 weeks	>34 weeks	• Fever (8/9)<br>• Cough (4/9)<br>• Cholecystitis (1/9)<br>• Diarrhoea (1/9)	• Preterm labour (6/9)	Unlikely (10)

Table 3. Infected neonatal symptoms and outcomes.

Author	Shah's Classification System for Maternal-Fetal-Neonatal SARS-CoV-2 Intrauterine Infections[11]	Details of mother of affected neonate	Details of affected neonate	Neonatal complications	1 min Apgar score	5 min Apgar score
Abasse et al.[12]	Unclassified – Possible (1)*			• Fever<br>• Respiratory difficulties<br>• NICU admission for monitoring	5	7
Alzamora et al.[14]	Unclassified – Possible(1)*	Mother possible 1: admitted at 33 weeks due to respiratory insufficiency. Was admitted to ICU for mechanical ventilation. Caesarean delivery due to decreased respiratory compromise.	Neonate possible: 33 weeks gestation, delivered by Caesarean section, required resuscitation and admitted to NICU for mechanical ventilation	• Cough<br>• Respiratory difficulties<br>• Lethargy<br>• NICU admission: mechanical ventilation	6	8
Demirjian et al.[18]	Unlikely (1)			• Fever<br>• Respiratory difficulties	5	9
Fenizia et al.[21]	Confirmed (1), Possible (1)	Mother confirmed 1: admitted at 33+6 with fever and dyspnoea, delivered spontaneously Mother confirmed 2: No reported symptoms, delivered spontaneously	Neonate confirmed 1: 34+4 weeks gestation, delivered spontaneous vaginal, asymptomatic, UA pH 7.12. Neonate possible 1: 39+2 weeks gestation, delivered spontaneous vaginal, asymptomatic, UA pH 7.14	• NICU admission for observation (2)	9 (2)	10 (2)
Govind et al.[23]	Possible (1)	Mother possible 1: admitted at 38 weeks with maternal pneumonia and respiratory distress.	Neonate possible 1: 38 weeks gestation, delivered by Caesarean section, required resuscitation and ECMO. Admitted to NICU for mechanical ventilation.	• Fever<br>• Cough<br>• Lethargy<br>• Foetal distress<br>• Respiratory difficulties<br>• NICU admission: mechanical ventilation	5	9
Hu et al.[24]	Unclassified – Possible (1)*			• Asymptomatic	8	9
Kirtsman et al.[28]	Probable (1)			• Vomiting/feeding difficulties<br>• Intermittent hypoglycaemic episodes<br>• NICU admission for observation	9	9
Knight et al.[29]	Unclassified – Probable (6)*, Possible (6)	• Maternal symptoms not reported.	• Six neonates tested positive within the first 12 h after birth.<br>• Two of the six infants with early onset SARS-CoV-2 infection were from unassisted vaginal births; four were born by caesarean, three of which were pre-labour.<br>• The six infants who	NICU admission (6/12)	NR	NR

(continued)

Table 3. Continued.

Author	Shah's Classification System for Maternal-Fetal-Neonatal SARS-CoV-2 Intrauterine Infections[11]	Details of mother of affected neonate	Details of affected neonate	Neonatal complications	1 min Apgar score	5 min Apgar score
			developed later infection were born by pre-labour caesarean (*n*=4) and vaginal birth (*n*=2). Only one of the infants with an early positive test for SARS-CoV-2 RNA was admitted to a neonatal unit for observation, compared with five infants with a later positive test.<br>• Symptoms were not reported			
Marzollo et al.[36]	Unclassified – Possible (1)*			• Respiratory difficulties<br>• Vomiting/feeding difficulties<br>• NICU admission: mechanical ventilation	9	10
Nie et al.[37]	Unlikely (1)			Asymptomatic	9	9
Oncel et al.[38]	Unlikely (4)		• Prematurity 2/4	• Fever (1/4)<br>• Cough (1/4)<br>• Respiratory difficulties (3/4)<br>• Vomiting/feeding difficulties (1/4)<br>• NICU admission: mechanical ventilation (1/4)	2 7 8 (2)	6 8 9 (2)
Patanè et al.[39]	Probable (2)			• Vomiting/feeding difficulties (2/2)	9 (2)	10 (2)
Patil et al.[40]	Possible (2)			Asymptomatic (2)	9	9
Sisman et al.[45]	Confirmed (1)			• Fever<br>• Respiratory difficulties	7	9
Vivanti et al.[46]	Confirmed (1)			• Foetal distress<br>• Neurological symptoms (hypertonia/ irritability)<br>• Resuscitation required<br>• NICU admission: mechanical ventilation	4	1
Wang et al.[47]	Unlikely (1)			• Vomiting/feeding difficulties	8	9
Wu et al.[48]	Unclassified – Possible (2)*, Unlikely (3)	Mother of neonate possible 1: presented with cough, had negative throat swab, clinically suspected with COVID-19. Mother of	Neonate Possible 1 (5): 40+4 weeks gestation, delivered by caesarean delivery, asymptomatic, admitted to NICU for observation Neonate Possible 2 (9): 39	• Fever (1/5)<br>• NICU admission for monitoring (2/5)	9 (2) 10 (3)	10 (5)

(continued)

Table 3. Continued.

Author	Shah's Classification System for Maternal-Fetal-Neonatal SARS-CoV-2 Intrauterine Infections[11]	Details of mother of affected neonate	Details of affected neonate	Neonatal complications	1 min Apgar score	5 min Apgar score
		neonate possible 2: asymptomatic, noted to have CT findings of bilateral focal GGO, negative throat swab, clinically suspected with COVID-19 Mother of neonate unlikely 1: asymptomatic, CT findings positive for left lung infectious lesions, bilateral pleural thickening and pleural effusion. Throat swab positive for Mother of Neonate Unlikely 2: Presented with fever, cough, vomiting with positive throat swab for Mother of Neonate Unlikely 3: Presented with fever, stuffy nose, shortness of breath, CT positive for bilateral scattered GGO, throat swab positive for	+1 weeks gestation, delivered by caesarean delivery, developed fever and admitted to NICU for observation Neonate Unlikely 1: 38+3 weeks gestation, delivered by cesarean delivery, asymptomatic, throat swab negative for Neonate Unlikely 2: 37+1 weeks gestation, delivered by caesarean delivery, asymptomatic, throat swab negative for Neonate Unlikely 3: 37+6 weeks gestation, delivered by caesarean delivery, asymptomatic, throat swab negative for			
Yu et al.[54]	Unlikely (3)		One neonate was found to have a mild pulmonary infection. The shortness of breath relieved quickly under neonatal care and was admitted to the NICU for monitoring	• Respiratory difficulties (1/3)<br>• NICU admission for monitoring (1/3)	9	10
Zamaniyan et al.[55]	Probable (1)			• Fever	8	9
Zeng et al.[56]	Unclassified – Possible (3)*		Neonate 1: 40 weeks gestation by caesarean delivery, developed lethargy and fever and required NICU admission. Neonate 2: 40+4 weeks gestation by caesarean delivery presenting with lethargy, vomiting, and fever. Neonate 3: 31+2 weeks gestation by caesarean delivery. Resuscitation was required. Apgar scores were 3, 4, and 5 at 1, 5, and 10 minutes after birth. Required NICU admission for mechanical ventilation with neonatal respiratory distress syndrome and pneumonia.	• Fever (2/3)<br>• Lethargy (2/3)<br>• Foetal distress (1/3)<br>• Respiratory difficulties (1/3)<br>• Vomiting/feeding difficulties (1/3)<br>• NICU admission for monitoring (2): mechanical ventilation (1)	3 (1), NR (2)	4 (1), NR (2)
